# A New Strategy to Uncover the Anticancer Mechanism of Chinese Compound Formula by Integrating Systems Pharmacology and Bioinformatics

**DOI:** 10.1155/2018/6707850

**Published:** 2018-07-18

**Authors:** Yifei Dai, Liang Sun, Weijie Qiang

**Affiliations:** ^1^Institute of Chinese Materia Medica, China Academy of Chinese Medical Sciences, Beijing, China; ^2^The China Institute for History of Medicine and Medical Literature, Beijing, China

## Abstract

Currently, cancer has become one of the major refractory diseases threatening human health. Complementary and alternative medicine (CAM) has gradually become an alternative choice for patients, which can be attributed to the high cost of leading cancer treatments (including surgery, radiotherapy, and chemotherapy) and the severe related adverse effects. As a critical component of CAM, traditional Chinese medicine (TCM) has increasing application in preventing and treating cancer over the past few decades. Huanglian Jiedu Decoction (HJD), a classical Chinese compound formula, has been recognized to exert a beneficial effect on cancer treatment, with few adverse effects reported. Nevertheless, the precise molecular mechanism remains unclear yet. In this study, we had integrated systems pharmacology and bioinformatics to explore the major active ingredients against cancer, targets for cancer treatment, and the related mechanisms of action. These targets were scrutinized using web-based Gene SeT Analysis Toolkit (WebGestalt), and 10 KEGG pathways were identified by enrichment analysis. Refined analysis of the KEGG pathways indicated that the anticancer effect of HJD showed a functional correlation with the p53 signaling pathway; moreover, HJD had potential therapeutic effect on prostate cancer (PCa) and small cell lung cancer (SCLC). Afterwards, genetic alterations and survival analysis of key targets for cancer treatment were examined in both PCa and SCLC. Our results suggested that such integrated research strategy might serve as a new paradigm to guide future research on Chinese compound formula. Importantly, such strategy contributes to studying the anticancer effect and the mechanisms of action of Chinese compound formula, which has also laid down the foundation for clinical application.

## 1. Introduction

According to a WHO report, cancer has become the leading killer of human health, which is associated with high recurrence rate and high mortality. Typically, the year 2012 has witnessed about 14 million new cancer cases and 8.2 million cancer-related deaths. It is estimated that the annual new cases will increase from 14 million to 22 million over the coming 20 years [1]. The existing anticancer treatments mainly include surgery, radiotherapy, and chemotherapy. However, the patients would eventually choose to discontinue the treatment due to the high cost of radiotherapy and chemotherapy, as well as the serious related adverse effects [2]. With the development of medicine, cancer is treated based on a comprehensive and diversified treatment, and complementary and alternative medicine (CAM) has become an alternative option for patients under such circumstances. Traditional Chinese medicine (TCM), a critical component of CAM, has been increasingly applied in preventing and treating cancer over the past few decades [3, 4]. As an adjuvant therapy, Chinese medicine shows beneficial effect on cancer treatment with few adverse effects reported [5].

Huanglian Jiedu Decoction (HJD), first recorded in the* Prescriptions for Emergent Reference (Zhouhou Beiji Fang)* written by Ge Hong, consists of four herbs, including* Coptidis Rhizoma *(Huanglian),* Scutellariae Radix* (Huangqin),* Phellodendri Chinrnsis Cortex* (Huangbo), and* Gardeniae Fructus* (Zhizi). HJD is a representative formula for cancer treatment, which is frequently employed to treat pancreatic cancer, breast cancer, liver cancer, and colorectal cancer (CRC) in clinical practice [6]. For instance, some results of pharmacological experiment suggest that HJD has anticancer effect on human liver cancer cells both in vitro and in vivo, which can also markedly extend the survival time of liver cancer bearing mice [7, 8]. However, the precise mechanism of its anticancer effect remains unclear so far.

**Figure 1:**
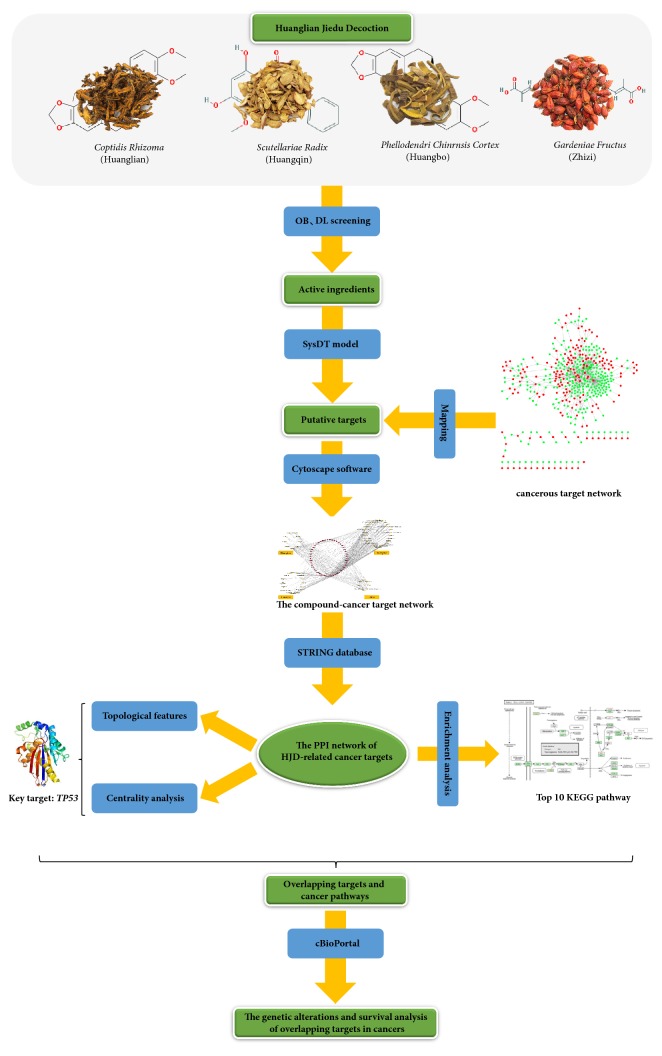
Integrated systems pharmacology and bioinformatics approach.

Chinese compound formula is characterized by the synergistic effects of multicomponent and multitarget. On this account, a method suitable for its characteristics is needed to reveal the underlying mechanism of action. Systems pharmacology is a new discipline studying the regularity and mechanism of drug-organism interaction at the system level [9]. It can study the changes in body function mechanisms caused by drug treatment for diseases from molecules, cells, tissues, to organs. Moreover, it would establish the interrelationships between drug efficacy and the organism at both microscopic levels (molecular and biochemical network levels) and macroscopic levels (tissue, organ, and overall levels). Besides, extremely abundant cancer data have been produced in recent years, with the rapid development of bioinformatics technology, including microarray, proteomics, and other high-throughput screening assays. By integrating systems pharmacology and bioinformatics, this study aimed to explore the relationships of HJD with its cancer-related targets and interactive genes and to reveal the underlying molecular mechanisms of action. Such strategy would be helpful for investigating the anticancer effect and the mechanism of action of Chinese compound formula, which could also provide the basis for clinical application. A flowchart of the research approach was presented in Figure 1. In addition, The Chinese herbal compound can be considered as a weak inhibitor with multicomponent and multitarget, and there are synergistic effects among multiple components. We hope to explore how this compound can actually work in the treatment of cancer, but it must be taken into account that the components of the compound are complex and not every component can play a role. Therefore, we screen out the main active components through multiple parameters and predict the targets of the active ingredients, so as to infer the therapeutic effect.

## 2. Materials and Methods

### 2.1. Construction of Cancerous Target Network and Chemical Component Database

All targets for cancer treatment could be accessed in DrugBank database (http://www.drugbank.ca/), and the cancerous target network was thereby constructed through Cytoscape [12]. In addition, HJD was comprised of four herbs, including* Coptidis Rhizoma* (Huanglian),* Scutellariae Radix* (Huangqin),* Phellodendri Chinrnsis Cortex* (Huangbo), and* Gardeniae Fructus* (Zhizi). All chemical components of these Chinese herbs had been collected into TcmSP [13], TcmID [14], TCM Database@Taiwan [15], and NCBI Pubchem databases and had been standardized to a constituent data supplemented in the TcmSP database. Finally, the number of chemical compounds in HJD was obtained, as shown in the Appendix.

### 2.2. Screening the Active Ingredients by OB Prediction

Oral bioavailability (OB) in vivo (%F), the unchanged fraction of the orally administered dose achieving systemic circulation, is one of the most commonly used pharmacokinetic parameters in drug screening cascades. In this study, a robust calculative system OBioavail 1.1 [16] was employed to predict the OB of the compounds, since it was difficult to assess the bioavailability of the complex TCM by “wet” experiments. It has combined the metabolism (cytochrome P450 3A4) and transporter (P-glycoproteins) information. Using this system, compounds with lower OB could be discarded, so that the amount of the original compounds could be distinctly reduced to a smaller set suitable for Chinese compound formulas. Compounds with the OB of ≥30% were selected as the active ingredients in this study. Such a threshold was selected based on (1) the use of a minimum number of components to maximally extract HJD information and (2) the fact that the obtained model could be reasonably explained by the reported pharmacological data.

### 2.3. Screening the Active Ingredients by Drug-Likeness Prediction

Before target prediction, some compounds considered chemically unsuitable for use were removed by drugs similarity index, which could be deduced as a delicate balance among the molecular properties affecting pharmacodynamics and pharmacokinetics, ultimately influencing its absorption, distribution, metabolism, and excretion (ADME) in human body like a drug. In this study, the drug-likeness (DL) index of a new compound was calculated according to the Tanimoto similarity [17].

(1) $$\begin{array}{c} f\left(\;A,B\;\right)=\frac{A\cdot B}{{\left|A\right|}^{2}+{\left|B\right|}^{2}-A\cdot B} \end{array}$$

where A represented the new compound and B stood for the average DL index of all the 6511 molecules in the DrugBank database based on the Dragon soft descriptors. Accordingly, molecules with the drug-likeness of <0.18 were also removed. Finally, compounds with both the OB of ≥30% and DL of ≥0.18 were considered as the active ingredients.

### 2.4. Prediction of the Targets of Active Ingredients

SysDT [18], the drug-target prediction model, was adopted to predict the targets of active ingredients. Briefly, SysDT was based on the 6511 drugs and 3987 targets of DrugBank database as well as the mutual correlation degree. Moreover, it was established using the stochastic forest algorithm and the support vector machine (SVM) algorithm, respectively. It turned out that the prediction model constructed by SVM was superior, with the consistency of 82.83%, sensitivity of 81.33%, and specificity of 93.62%. Using such model, targets with the SVM of > 0.7 were predicted as the putative targets of active ingredients. In addition, target information was integrated from SEA [19], STITCH [20], TTD [21], and HIT [22] databases to supplement this predictive model. Moreover, information regarding the physiological functions of all targets was obtained from the TTD and UniProt databases.

### 2.5. Construction of the Network and Topological Analysis

Associations between active ingredients and putative targets were constructed into the compound-target network of HJD using Cytoscape v3.4.0 software [12], which was then mapped with the cancerous target network to obtain the compound-cancer target network of HJD, including all HJD-related targets for cancer treatment. Afterwards, the protein-protein interaction (PPI) network of HJD-related targets for cancer treatment was constructed by STRING [23]. Subsequently, topology analysis was performed using the Network Analyzer plug-in to output the main topological parameters of this network [24].

### 2.6. Screening Key Targets and KEGG Pathway Enrichment Analysis

The centrality algorithm is a key method to measure the importance degree of nodes in the whole network, with a larger value indicating a higher importance degree of node in the whole network and greater influence on the structure and function of the whole network. In this study, the degree centrality algorithm was adopted as the major algorithm, supplemented by the closeness centrality and the betweenness centrality algorithm, so as to select and evaluate the key anticancer targets of HJD. Additionally, the biological information and attribution embedded in the anticancer targets were then analyzed using a web-based integrated data mining system, WebGestalt [25]. Biochemical pathways and functions linked to the anticancer targets of HJD were specifically queried and navigated by the KEGG pathway enrichment analysis tool in WebGestalt. Eventually, the top 10 pathways with an adjusted P value of <0.01 were selected.

### 2.7. Exploration of the Cancer Genomics Data Linked to HJD by cBio Cancer Genomics Portal

The cBio Cancer Genomics Portal (http://cbioportal.org), an open platform to explore the multidimensional cancer genomics data, can encapsulate the molecular profiling data obtained from cancer tissues and cell lines into the readily understandable genetic, epigenetic, gene expression, and proteomic events [26]. Specifically, the complex cancer genomics profiles can be easily accessed using the query interface of the Portal, which enables the researchers to explore and compare the genetic alterations across samples. Furthermore, the obtained underlying data can thereby be linked to clinical outcomes, which has facilitated the novel discovery in biological systems.

In this study, the cBio Portal was utilized to examine the connectivity of HJD-related targets for cancer treatment across all studies on PCa and SCLC available in the databases. These targets in all sample studies on PCa and SCLC were classified as altered or nonaltered using the Portal search function. The genomics datasets were then presented using OncoPrint as the heatmap, a visually appealing display of alterations in microarrays across cancer samples [27]. Another feature of the Portal was that, it could generate multiple visualization platforms through grouping PCa abd SCLC-associated alterations using the input from key HJD-related targets for cancer treatment [27–31]. In the meantime, the survival of these targets in PCa and SCLC was analyzed using survival option embedded in the Portal, a tool integrating the survival Kaplan-Meier estimate and the survival data in the TCGA database.

## 3. Results

### 3.1. Screening the Active Ingredients and Visualization of the Compound-Cancer Target Network

**Figure 2:**
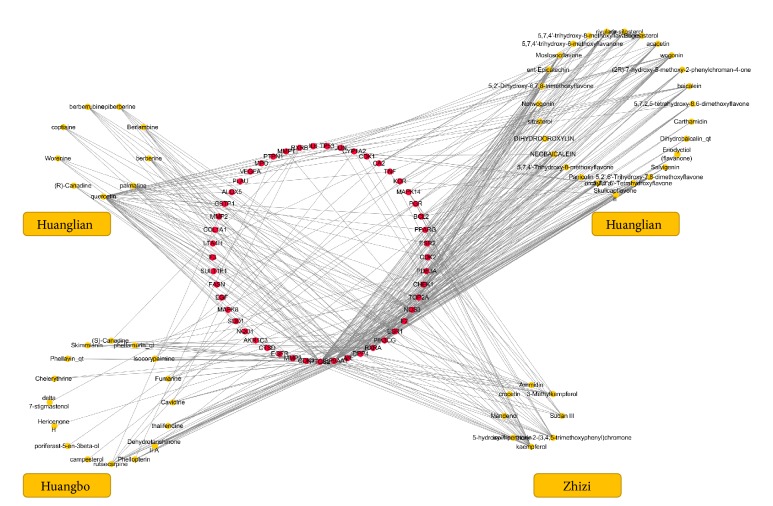
The compound-cancer target network of HJD. The yellow nodes represented active ingredients, while the red ones stood for anticancer targets.

Compounds contained in all 4 herbs constituting HJD were collected through several databases, including Huanglian (48), Huangqin (143), Huangbo (140), and Zhizi (98). A total of 85 compounds with OB of ≥ 30% and DL of ≥ 0.18 were identified, among which only 59 active ingredients targeting the anticancer targets were screened (the Appendix). Correlations of the active ingredients with their anticancer targets were visualized through Cytoscape, and the compound-cancer target network was also obtained for subsequent analysis (Figure 2).

### 3.2. Construction of the PPI Network of HJD-Related Targets for Cancer Treatment as well as Topological Analysis

**Figure 3:**
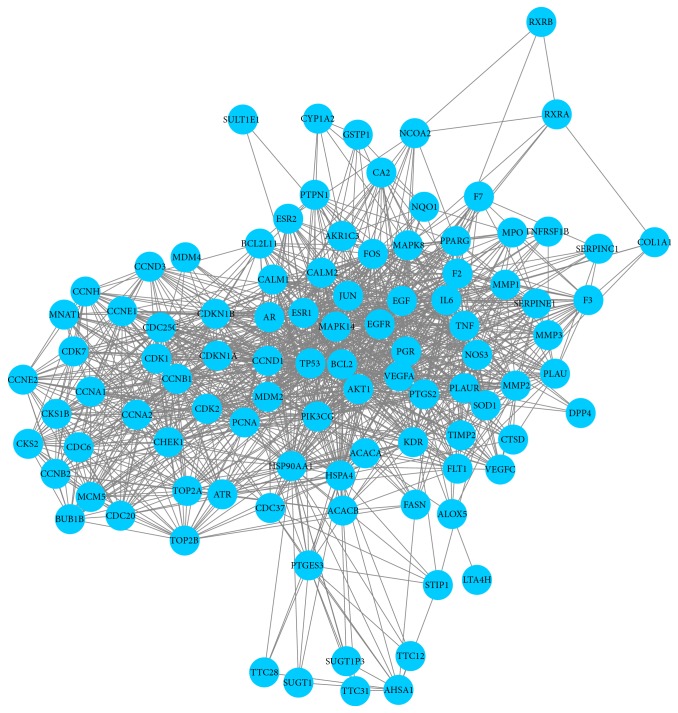
The PPI network of HJD-related targets for cancer treatment. The blue nodes represented HJD-related targets, while the edges represented the interaction between targets.

The HJD-related targets for cancer treatment could be obtained through the compound-cancer target network. In addition, the “protein-protein interaction (PPI) option” embedded in STRING was also adopted for further analysis, and a PPI network containing 98 interactive targets was also identified (Figure 3). Later, the topological features of this network were calculated with the Network Analyzer plug-in (Table 1), which consisted of an entire portion of the interaction between the anticancer targets, with an average number of direct neighbors of 20.959. Besides, the degree of some nodes was much higher than the average number of direct neighbors. In the degree centrality algorithm, a higher degree of a node indicated greater impact on the whole network. In this network, the degree distribution between nodes was uneven. These nodes, which were twice the average number of direct neighbors, were then define as Hub nodes in this study, indicating their importance in the network for subsequent investigation.

### 3.3. Searching and Analysis of the Key Targets

Three centrality algorithms were employed for key target screening, including degree centrality, closeness centrality, and betweenness centrality. Of them, the closeness centrality algorithm has emphasized the average shortest path length between nodes and other nodes. In contrast, the betweenness centrality algorithm measures the number of nodes on the shortest path of other nodes, which suggests the frequency that the shortest path between the other nodes passes through one node. In other words, if the shortest path of the other nodes often passes through this node, then this node shows a high importance or ability, which can modulate information transmission of other nodes as a link between the other nodes.

These 3 algorithms were used to calculate the whole network, and the top 30 targets were summarized based on the algorithm results, as shown in Table 2. Consistently, nodes that were twice the average number of direct neighbors were defined as Hub nodes, including* TP53*,* AKT1*,* EGF*,* PCNA*,* JUN*,* VEGFA*,* ESR1*, and* IL6*. It should be noted that* TP53* ranked the top among the three centrality algorithms, indicating that the primary target pathway under control or mediated by HJD was associated with* TP53*. In addition,* AKT1* took up the second place, which was only second to* TP53*. As a critical component in the PI3K-AKT signaling pathway,* AKT1* was closely correlated with the occurrence and development of human cancers. Baicalin and baicalein, the main active ingredients of Huangqin, had been reported to show a definite relationship with the downregulation of the PI3K-AKT pathway in anticancer effect [23, 24]. Consequently, the* AKT1*-related signaling pathway might also have an important link with the anticancer effect of HJD.

### 3.4. Analysis of the KEGG Pathway

To explore the biological mechanism underlying the anticancer effect of HJD, the KEGG pathway enrichment analysis embedded in WebGestalt was performed. Typically, the top 10 KEGG pathways linked to all targets in the PPI network were obtained, including cell cycle (24), pathways in cancer (31), the p53 signaling pathway (15), the AGE-RAGE signaling pathway in diabetic complications (17), prostate cancer (16), endocrine resistance (16), hepatitis B (18), the PI3K-Akt signaling pathway (25), small cell lung cancer (14), and the FoxO signaling pathway (15) (Table 3). Broad grouping of the KEGG pathway analysis suggested that the anticancer effect of HJD was closely correlated with the following cancer-related signaling pathways with potential mechanisms, including (1) control of cancer cell proliferation and survival by p53-mediated cell cycle control, (2) the PI3K-Akt signaling pathway regulating the growth, proliferation, and invasion and metastasis of cancer cells by mediating the FoxO signaling pathway, and (3) the potential treatment of breast cancer achieved through regulating endocrine resistance.* TP53* ranked the top among the 3 centrality algorithms (Table 2); as a result, emphasis was directed to the p53 signaling pathway. The KEGG analysis results probably indicated that the anticancer effect of HJD showed a functional correlation with* TP53*. In addition, the enrichment KEGG pathway analysis also suggested that 16 and 14 targets were associated with PCa and SCLC, respectively (Table 3).

### 3.5. Mining the Genetic Alterations and Survival Analysis

It had been proved that HJD displayed therapeutic effects on different cancers; however, its specific biological mechanisms remained unclear so far. KEGG enrichment analysis revealed that HJD was correlated with the cancer-related pathways (Table 3). To further explore the validity of such correlation, cBio Portal, a web-based integrated data mining system, was adopted to examine the genetic alterations and survival analysis associated with HJD-related targets in PCa and SCLC. The p53 signaling pathway was the main target of HJD; consequently, the overlapping targets of the p53 signaling pathway with PCa and SCLC were studied. The results discovered that 8 overlapping targets were associated with the KEGG assay embedded in WebGestalt, including 7 in PCA (*CDK2, CDKN1A, MDM2, CCND1, TP53, CCNE1,* and* CCNE2*) and 5 in SCLC (*CDK2, CCND1, TP53, CCNE1,* and* CCNE2*). Therefore, the genomic and clinical characteristics of these targets in PCa and SCLC were examined, respectively (Table 2).

**Figure 4:**
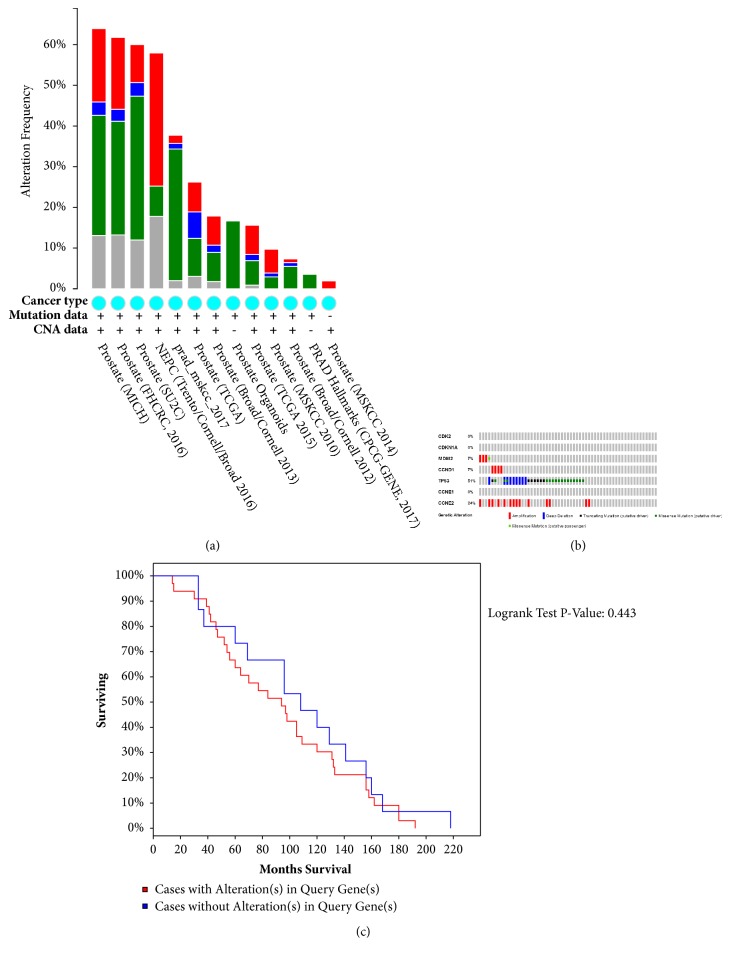
The genetic alterations and survival analysis related to 7 overlapping targets (including CDK2, CDKN1A, MDM2, CCND1, TP53, CCNE1, and CCNE2) in PCa studies embedded in cBio cancer genomics Portal. (a) Overview of changes in 7 overlapping targets in genomics datasets available in 13 different PCa studies. (b) OncoPrint: a visual summary of alterations across a set of prostate samples (data taken from the Michigan studies, Nature 2012) [10] based on a query of the 7 overlapping targets. Distinct genomic alterations including mutations and copy number alterations (CNAs, exemplified by gene amplifications and homozygous deletions) were summarized, and the color codes represented % changes) in particular targets in individual cancer samples. Each row stood for a gene, and each column represented a cancer sample. Red bars stood for gene amplifications, blue bars represented homozygous deletions, and green squares indicated nonsynonymous mutations. (c) K-M curve between groups with alterations and without alterations. Red line represented cases with alterations, and the blue one indicated cases without. The X-axis was overall survival (OS, months), and the Y-axis stood for the survival rate. Kaplan-Meier test was performed.

13 studies on PCa were analyzed [10, 32–40], the results of which indicated 1.9% to 63.9% alterations in the gene sets/pathways submitted for analysis (Figure 4(a)). Multiple genetic alterations observed across each set of cancer samples from the Michigan study [10] with the most significant genomic changes were summarized and presented using OncoPrint. The results indicated that 37 cases (63%) had an alteration in at least one of the 7 targets, and the alteration frequency in each of the selected targets was presented in Figure 4(b).* CDK2, CDKN1A,* and* CCNE1* were not associated with genetic alterations. For* MDM2, CCND1,* and* CCNE2*, most alterations were classified as amplification.* TP53-*associated genetic alterations included deep deletions and missense/truncating mutations. The alterations in these targets showed a cooccurrence trend across samples. However, mutual exclusivity analysis revealed no statistical significance (p=0.183) (data not shown). More interestingly, cases with genetic alterations were linked with a poorer survival compared with those without alterations (P=0.443, Figure 4(c)).

**Figure 5:**
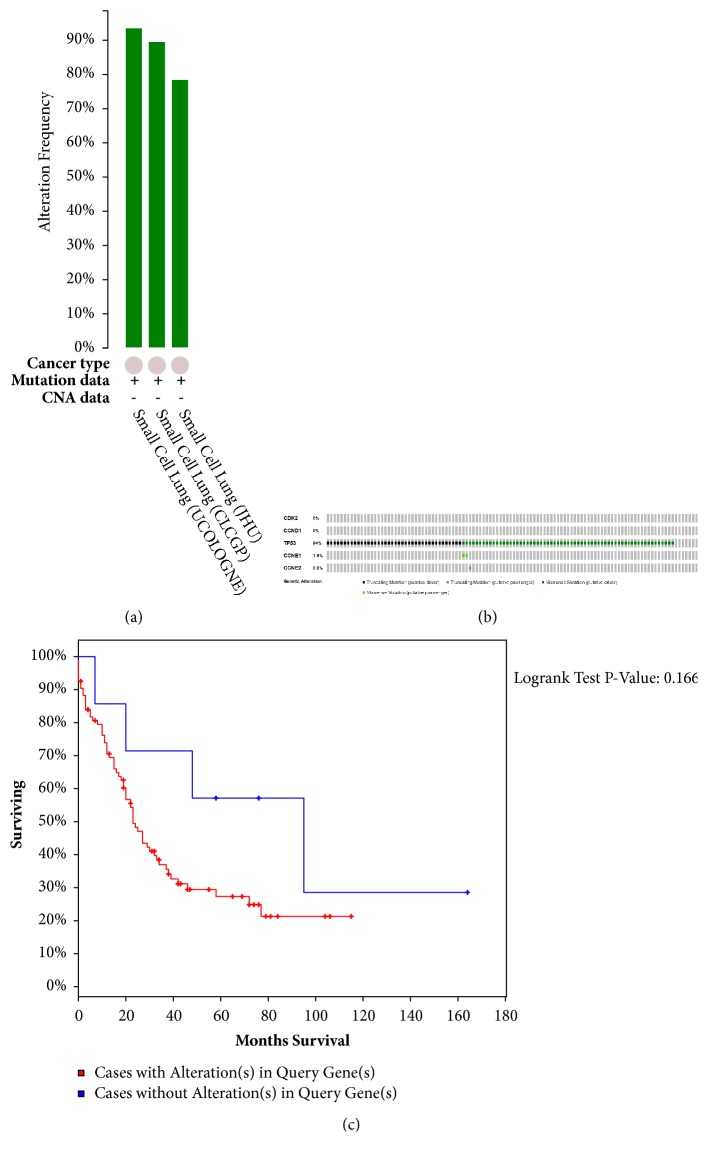
The genetic alterations and survival analysis related to the 5 overlapping targets (including CDK2, CCND1, TP53, CCNE1, and CCNE2) in SCLC studies embedded in cBio cancer genomics Portal. (The annotations were consistent with those in Figure 4.) (a) Overview of changes in 5 overlapping targets in genomics datasets available in 3 different SCLC studies. (b) OncoPrint (data taken from the U Cologne studies, Nature 2015) [11] based on a query of the 5 overlapping targets). (c) K-M curve between groups with alterations and without alterations.

Among the 3 SCLC studies analyzed [11, 41, 42], 78.6% to 93.6% alterations were found in the gene sets/pathways submitted for analysis (Figure 5(a)). Multiple genetic alterations observed across each set of cancer samples from the U Cologne study with the most significant genomic changes were summarized and presented using OncoPrint [11]. The results indicated that 103 cases (94%) had an alteration in at least one of the 5 targets, and the alteration frequency in each of the selected targets was shown in Figure 5(b). Different from results of PCa study, these results indicated that almost all genetic alterations occurred in TP53, whereas no genetic alterations were seen in CDK2 or CCND1. CCNE1-associated genetic alterations were classified as missense mutations, while CCNE2-associated ones were classified as truncating mutations. In comparison, TP53-associated genetic alterations included both missense mutations and truncating mutations. The mutual exclusivity analysis still displayed no statistical significance (p = 0.876) (data not shown). More interestingly, cases with genetic alterations also had a poorer survival relative to those without (P=0.166, Figure 5(c)).

## 4. Discussion

HJD serves as the object of study in this work. To elucidate the anticancer molecular mechanism of HJD, we have integrated systems pharmacology and bioinformatics. As a result, a number of public databases as the research basis and a set of tools are available to elucidate the molecular mechanisms and the relationship with the clinical outcomes of cancers. 3 steps are carried out in our workflow. (i) The cancerous target network is constructed through the DrugBank database, and all chemical components contained in the 4 medicines are obtained by databases, such as TcmSP, TcmID, TCM Database@Taiwan, and NCBI Pubchem. Subsequently, the active ingredients are screened based on the criteria of OB of ≥30% and DL of ≥0.18, and the targets of these active ingredients were then predicted using the SysDT model. Ultimately, 59 anticancer active ingredients and their anticancer targets were identified by mapping with the cancerous target network (the Appendix). (ii) Based on these anticancer targets, a PPI network containing 98 targets is constructed by STRING (Figure 2), and topological analysis is therefore performed. Eight key anticancer targets (including TP53, AKT1, EGF, PCNA, JUN, VEGFA, ESR1, and IL6) are screened through the topological parameters (Table 1) and 3 centrality algorithms (Table 2). Afterwards, the top 10 KEGG pathways are identified by enrichment analysis of the 98 targets (Table 3). (iii) Taking TP53 as the main object of study, we have compared the p53 signaling pathway between PCa and SCLC, and 8 overlapping targets are obtained. Then, the genetic alterations and survival analysis of the overlapping targets in PCa and SCLC are performed, so as to evaluate the relevance of the p53 signaling pathway with HJD in treating cancer.

HJD has been suggested in a report to inhibit angiogenesis through suppressing the expression of VEGFA and MMP-9, thus further restraining cancer growth [43]. Similarly, we also discover that VEGFA is a key target in the anticancer activity of HJD using network analysis (Table 2). In addition, a study shows that HJD can obviously inhibit the proliferation of human SCLC NCI-H446 cells [44]. Coincidently, our findings also support that HJD has certain therapeutic effect on SCLC, which is probably achieved through regulating the p53 signaling pathway. However, no other related literature reports that HJD has therapeutic effect on PCa, which may account for a future research direction pending further validation of the experiment. Interestingly, we find through KEGG enrichment analysis that the AGE-RAGE signaling pathway is also present in diabetic complications. The therapeutic effect of HJD on diabetes and its complications has been approved in lots of literature; nonetheless, no existing study indicates HJD works through this pathway. Therefore, it remains to be further studied whether the AGE-RAGE signaling pathway may be a potential mechanism of HJD in treating diabetes and its complications.

Compared with studies integrating systems pharmacology and network pharmacology, the current study has a certain biological rationality, since it has bridged HJD to its target genes and linked it with biological effects. Moreover, this study has also illustrated the relationship between the molecular mechanism of HJD and the clinical outcome of cancer through a set of network-based tools. This approach is greatly different from the use of experimental techniques to prove a few relationships at a time; instead, it can reduce redundant experiments from different laboratories. The use of such a new research strategy may remarkably contribute to (i) understanding the molecular biological mechanisms of Chinese compound formula, (ii) revealing the primary effects and targets of HJD on cancers, and (iii) promoting the clinical use of Chinese compound formula and laying down the clinical foundation. This method can be used not only in the study on HJD, but also on other Chinese compound formulas and on medicine combination therapy.

However, there are some shortcomings deserving our attention. The compounds contained in the herbal medicines are obtained based on databases; therefore, the quality of databases would directly affect the final compounds obtained. Moreover, the selection of screening parameters and the setting of threshold can also affect the number of active ingredients obtained. All of these may influence the final analysis.

In conclusion, the targets of HJD will undoubtedly be confirmed thanks to a growing number of studies on HJD carried out using traditional experimental techniques and methods. However, the relationship with the biological effects of HJD remains unclear yet. We believe that the use of this method can help to offset some uncertainties of HJD related to its target and its subsequent phenotypic expression. Furthermore, this approach contributes to determining the feasibility of future experiments. In the future, molecular biology experiments about the key targets and pathways of HJD can be carried out on the basis of the current study. Apart from PCa and SCLC, many studies have also reported the antitumor effect of HJD on other tumors, such as lung cancer, liver cancer, breast cancer, and colon cancer. These findings reveal that it remains to be further studied whether the connectivity between HJD and PCa as well as SCLC can be extended to other cancers.

## Figures and Tables

**Table 1:** The topological features of the PPI network.

Parameters	numerical value	Parameters	numerical value
Clustering coefficient	0.667	Number of nodes	98
Connected components	1	Number of edges	1027
Network diameter	4	Network density	0.216
Network radius	2	Network heterogeneity	0.676
Network centralization	0.474	Isolated nodes	0
Shortest paths	9506(100%)	Number of self-loops	0
Characteristic path length	2.006	Multiedge node pairs	0
Avg. number of neighbors	20.959	-	-

**Table 2:** The centrality analysis of PPI network of HJD-related cancer targets.

Name	Degree	Name	Betweenness Centrality	Name	Closeness Centrality
*TP53*	66	*TP53*	0.12965202	*TP53*	0.75193798
*AKT1*	49	*HSP90AA1*	0.07551998	*AKT1*	0.65986395
*EGF*	47	*HSPA4*	0.06902023	*EGF*	0.65100671
*PCNA*	47	*IL6*	0.06776382	*VEGFA*	0.64666667
*JUN*	46	*AKT1*	0.04867524	*PCNA*	0.64666667
*VEGFA*	44	*VEGFA*	0.04389668	*JUN*	0.64666667
*ESR1*	42	*PCNA*	0.03662677	*IL6*	0.63815789
*IL6*	42	*EGF*	0.034913	*ESR1*	0.62987013
*CDK1*	41	*ESR1*	0.02917485	*BCL2*	0.62580645
*BCL2*	41	*JUN*	0.02822688	*EGFR*	0.62179487
*HSP90AA1*	40	*TNF*	0.02701286	*HSP90AA1*	0.61783439
*CDK2*	40	*CDK1*	0.02175952	*TNF*	0.61783439
*CCND1*	40	*PTGS2*	0.02156667	*CDKN1A*	0.61006289
*EGFR*	40	*PPARG*	0.02083383	*FOS*	0.61006289
*TNF*	39	*AKR1C3*	0.02075601	*PIK3CG*	0.61006289
*CDKN1A*	38	*ALOX5*	0.02061856	*HSPA4*	0.60625
*PIK3CG*	37	*AR*	0.01863776	*CDK2*	0.60625
*FOS*	37	*CDK2*	0.01810019	*AR*	0.60248447
*HSPA4*	37	*NOS3*	0.017597	*MAPK8*	0.60248447
*MAPK8*	35	*EGFR*	0.01749873	*PTGS2*	0.59876543
*AR*	34	*CCND1*	0.01665403	*CDK1*	0.59509202
*CCNB1*	33	*MAPK8*	0.01587777	*CCND1*	0.59509202
*NOS3*	33	*CDKN1A*	0.01556023	*NOS3*	0.59146341
*PTGS2*	33	*BCL2*	0.0151564	*MMP2*	0.58083832
*CHEK1*	30	*FOS*	0.01323593	*MAPK14*	0.58083832
*CDKN1B*	30	*MMP2*	1.25E-02	*CALM2*	0.57058824
*MMP2*	30	*PGR*	9.72E-03	*CALM1*	0.57058824
*MAPK14*	30	*CDKN1B*	9.65E-03	*CDKN1B*	0.56725146
*CCNA2*	29	*PIK3CG*	9.52E-03	*MDM2*	0.56725146
*MDM2*	29	*CALM2*	9.18E-03	*KDR*	0.55747126

**Table 3:** KEGG pathway analysis.

Pathway Name	#Gen	Uniprot name (corresponding gene set)	Statistics
Cell cycle	24	CDK2 CDK7 CDKN1A CDKN1B CHEK1 MCM5 MDM2 PCNA ATY CCND1 BUB1B TP53 CCNA2 CCNA1 CCNB1 CCND3 CCNE1 CCNH CCNB2 CCNE2 CDK1 CDC6 CDC20 CDC25C	C=124; O=24; E=1.59; R=15.09; rawP=0e+00; adjP=0e+00
Pathways in cancer	31	CDK2 CDKN1A CDKN1B CKS1B CKS2 EGF EGFR AKT1 FOS GSTP1 HSP90AA1 IL6 AR JUN MDM2 MMP1 MMP2 PIK3CG PPARG MAPK8 PTGS2 CCND1 BCL2 RXRA RXRB TP53 VEGFA VEGFC CCNA1 CCNE1 CCNE2	C=397; O=31; E=5.09; R=6.09; rawP=0e+00; adjP=0e+00
p53 signaling pathway	15	CDK2 CDKN1A CHEK1 MDM2 MDM4 SERPINE1 ATR CCND1 TP53 CCNB1 CCND3 CCNE1 CCNB2 CCNE2 CDK1	C=69; O=15; E=0.88; R=16.95; rawP=4.22e-15; adjP=3.78e-13
AGE-RAGE signaling pathway in diabetic complications	17	CDKN1B COL1A1 MAPK14 AKT1 F3 IL6 JUN MMP2 NOS3 SERPINE1 PIK3CG MAPK8 CCND1 BCL2 TNF VEGFA VEGFC	C=101; O=17; E=1.3; R=13.13; rawP=5e-15; adjP=3.78e-13
Prostate cancer	16	CDK2 CDKN1A CDKN1B EGF EGFR AKT1 GSTP1 HSP90AA1 AR MDM2 PIK3CG CCND1 BCL2 TP53 CCNE1 CCNE2	C=89; O=16; E=1.14; R=14.02; rawP=1.15e-14; adjP=7e-13
Endocrine resistance	16	CDKN1A CDKN1B MAPK14 EGFR AKT1 ESR1 ESR2 FOS JUN MDM2 MMP2 PIK3CG MAPK8 CCND1 BCL2 TP53	C=98; O=16; E=1.26; R=12.73; rawP=5.64e-14; adjP=2.85e-12
Hepatitis B	18	CDK2 CDKN1A CDKN1B AKT1 FOS IL6 JUN PCNA PIK3CG MAPK8 CCND1 BCL2 TNF TP53 CCNA2 CCNA1 CCNE1 CCNE2	C=146; O=18; E=1.87; R=9.62; rawP=2.03e-13; adjP=8.77e-12
PI3K-Akt signaling pathway	25	BCL2L11 CDK2 CDKN1A CDKN1B CDC37 COL1A1 EGF EGFR AKT1 FLT1 HSP90AA1 IL6 KDR MDM2 NOS3 PIK3CG CCND1 BCL2 RXRA TP53 VEGFA VEGFC CCND3 CCNE1 CCNE2	C=341; O=25; E=4.37; R=5.72; rawP=4.23e-13; adjP=1.6e-11
Small cell lung cancer	14	CDK2 CDKN1B CKS1B CKS2 AKT1 PIK3CG PTGS2 CCND1 BCL2 RXRA RXRB TP53 CCNE1 CCNE2	C=86; O=14; E=1.1; R=12.7; rawP=2.52e-12; adjP=8.48e-11
FoxO signaling pathway	15	BCL2L11 CDK2 CDKN1A CDKN1B MAPK14 EGF EGFR AKT1 IL6 MDM2 PIK3CG MAPK8 CCND1 CCNB1 CCNB2	C=134; O=15; E=1.72; R=8.73; rawP=1.03e-10; adjP=3.12e-09

The following statistics were listed in the row: C: the number of reference targets in the category; O: the number of targets in both the gene set and the category; E: the expected number in the category; R: ratio of enrichment; rawP: p value upon hypergeometric test; and adjP: p value adjusted by the multiple test adjustment.

**Table 4:** Anticancer active ingredients, oral bioavailability (OB), and drug-likeness (DL) of HJD.

Name	Active Ingredient	Chemical Structure	OB/%	DL
Huanglian	(R)-Canadine	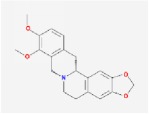	55.37	0.77
	berberine	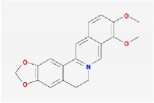	36.86	0.78
	berberrubine	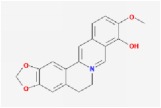	35.74	0.73
	Berlambine	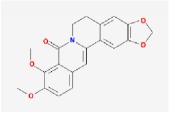	36.68	0.82
	coptisine	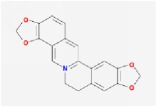	30.67	0.86
	epiberberine	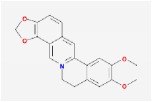	43.09	0.78
	palmatine	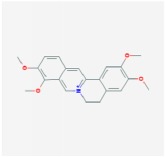	64.6	0.65
	quercetin	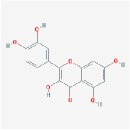	46.43	0.28
	Worenine	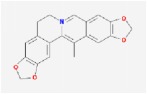	45.83	0.87

Huangqin	(2R)-7-hydroxy-5-methoxy-2-phenylchroman-4-one	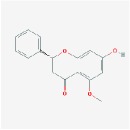	55.23	0.2
	5,2′,6′-Trihydroxy-7,8-dimethoxyflavone	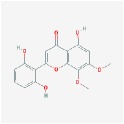	45.05	0.33
	5,2′-Dihydroxy-6,7,8-trimethoxyflavone	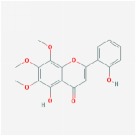	31.71	0.35
	5,7,2,5-tetrahydroxy-8,6-dimethoxyflavone	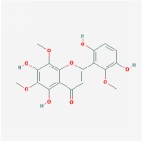	33.82	0.45
	5,7,2′,6′-Tetrahydroxyflavone	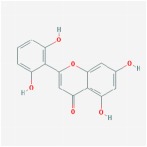	37.01	0.24
	5,7,4′-trihydroxy-6-methoxyflavanone	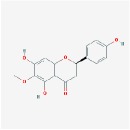	36.63	0.27
	5,7,4′-trihydroxy-8-methoxyflavanone	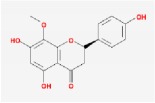	74.24	0.26
	5,7,4′-Trihydroxy-8-methoxyflavone	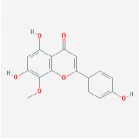	36.56	0.27
	acacetin	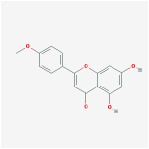	34.97	0.24
	baicalein	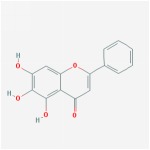	33.52	0.21
	beta-sitosterol	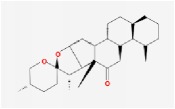	36.91	0.75
	Carthamidin	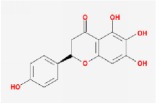	41.15	0.24
	Dihydrobaicalin_qt	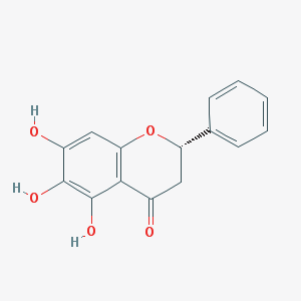	40.04	0.21
	Dihydrooroxylin	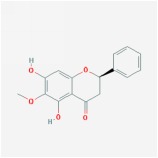	66.06	0.23
	ent-Epicatechin	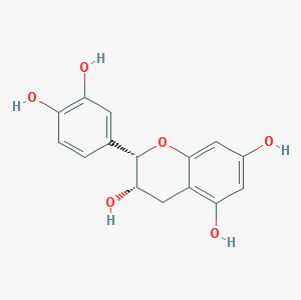	48.96	0.24
	Eriodyctiol (flavanone)	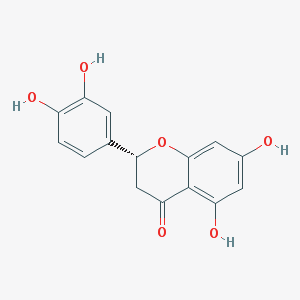	41.35	0.24
	Moslosooflavone	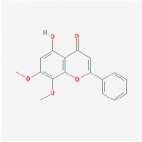	44.09	0.25
	NEOBAICALEIN	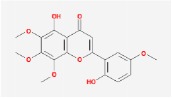	104.34	0.44
	Norwogonin	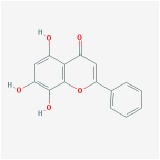	39.4	0.21
	oroxylin a	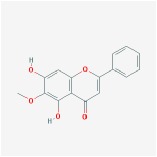	41.37	0.23
	Panicolin	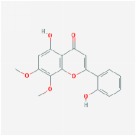	76.26	0.29
	rivularin	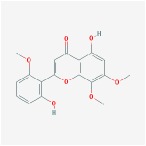	37.94	0.37
	Salvigenin	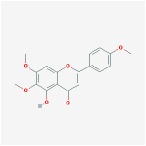	49.07	0.33
	sitosterol	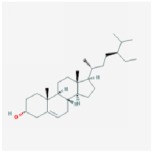	36.91	0.75
	Skullcapflavone II	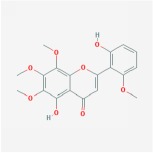	69.51	0.44
	Stigmasterol	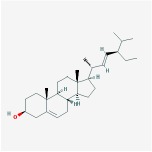	43.83	0.76
	wogonin	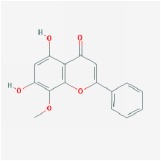	30.68	0.23

Huangbo	(S)-Canadine	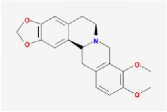	53.83	0.77
	campesterol	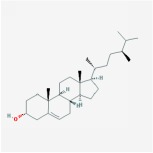	37.58	0.71
	Cavidine	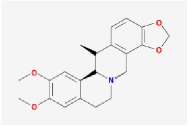	35.64	0.81
	Chelerythrine	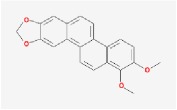	34.18	0.78
	Dehydrotanshinone II A	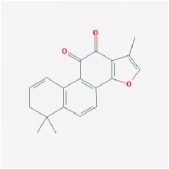	43.76	0.4
	delta 7-stigmastenol	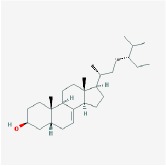	37.42	0.75
	Fumarine	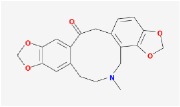	59.26	0.83
	Hericenone H	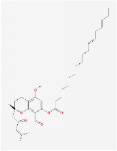	39	0.63
	Isocorypalmine	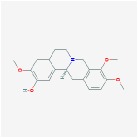	35.77	0.59
	phellamurin_qt	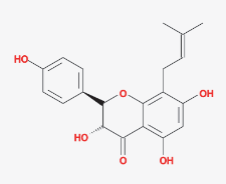	56.6	0.39
	Phellavin_qt	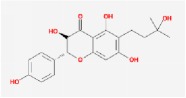	35.86	0.44
	Phellopterin	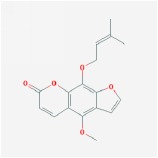	40.19	0.28
	poriferast-5-en-3beta-ol	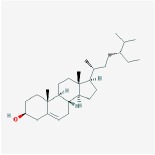	36.91	0.75
	rutaecarpine	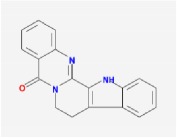	40.3	0.6
	Skimmianin	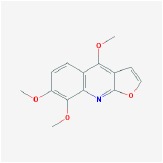	40.14	0.2
	thalifendine	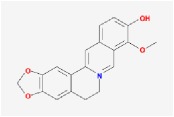	44.41	0.73

Zhizi	3-Methylkempferol	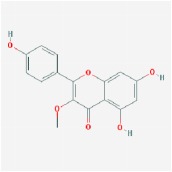	32.03	0.76
	5-hydroxy-7-methoxy-2-(3,4,5-trimethoxyphenyl) chromone	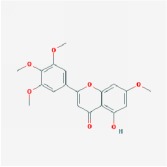	34.55	0.22
	Ammidin	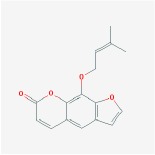	84.07	0.59
	crocetin	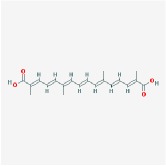	36.91	0.75
	isoimperatorin	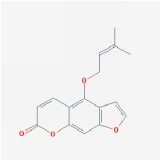	42	0.19
	kaempferol	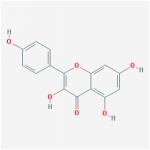	33.55	0.42
	Mandenol	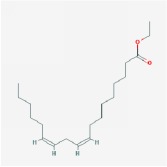	45.46	0.23
	Sudan III	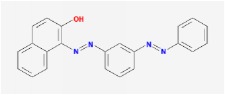	60.16	0.26

## Data Availability

The data used to support the findings of this study are available from the corresponding author upon request.

## Appendix

See Table 4.

## References

[1] Hullmann S. E., Robb S. L., Rand K. L. (2016). Life goals in patients with cancer: A systematic review of the literature. *Psycho-Oncology*.

[2] Tao J. J., Visvanathan K., Wolff A. C. (2015). Long term side effects of adjuvant chemotherapy in patients with early breast cancer. *The Breast Journal*.

[3] Zhong L. L. D., Chen H.-Y., Cho W. C. S., Meng X.-M., Tong Y. (2012). The efficacy of Chinese herbal medicine as an adjunctive therapy for colorectal cancer: a systematic review and meta-analysis. *Complementary Therapies in Medicine*.

[4] Zhu L., Li L., Li Y., Wang J., Wang Q. (2016). Chinese Herbal Medicine as an Adjunctive Therapy for Breast Cancer: A Systematic Review and Meta-Analysis. *Evidence-Based Complementary and Alternative Medicine*.

[5] Qi F., Zhao L., Zhou A. (2015). The advantages of using traditional Chinese medicine as an adjunctive therapy in the whole course of cancer treatment instead of only terminal stage of cancer. *Bioscience Trends*.

[6] Sun J., Wen Q. H., Song Y. (2006). Study on antitumor activities of huanglian jiedu decoction. *China Journal of Chinese Materia Medica*.

[7] Wang N., Feng Y., Tan H. Y. (2015). Inhibition of eukaryotic elongation factor-2 confers to tumor suppression by a herbal formulation Huanglian-Jiedu decoction in human hepatocellular carcinoma. *Journal of Ethnopharmacology*.

[8] Hsu Y. L., Kuo P. L., Tzeng T. F. (2008). Huang-lian-jie-du-tang, a traditional Chinese medicine prescription, induces cell-cycle arrest and apoptosis in human liver cancer cells *in vitro* and *in vivo*. *Journal of Gastroenterology and Hepatology*.

[9] Zhao S., Iyengar R. (2012). Systems pharmacology: Network analysis to identify multiscale mechanisms of drug action. *Annual Review of Pharmacology and Toxicology*.

[10] Grasso C. S., Wu Y.-M., Robinson D. R. (2012). The mutational landscape of lethal castration-resistant prostate cancer. *Nature*.

[11] George J., Lim J. S., Jang S. J. (2015). Comprehensive genomic profiles of small cell lung cancer. *Nature*.

[12] Shannon P., Markiel A., Ozier O. (2003). Cytoscape: a software Environment for integrated models of biomolecular interaction networks. *Genome Research*.

[13] Ru J., Li P., Wang J. (2014). TCMSP: a database of systems pharmacology for drug discovery from herbal medicines. *Journal of Cheminformatics*.

[14] Xue R., Fang Z., Zhang M., Yi Z., Wen C., Shi T. (2013). TCMID: Traditional Chinese medicine integrative database for herb molecular mechanism analysis. *Nucleic Acids Research*.

[15] Chen C. Y.-C. (2011). TCM Database@Taiwan: the world's largest traditional Chinese medicine database for drug screening in silico. *PLoS ONE*.

[16] Xu X., Zhang W., Huang C. (2012). A novel chemometric method for the prediction of human oral bioavailability. *International Journal of Molecular Sciences*.

[17] Yang H., Zhang W., Huang C. (2014). A novel systems pharmacology model for herbal medicine injection: A case using reduning injection. *BMC Complementary and Alternative Medicine*.

[18] Yu H., Chen J., Xu X. (2012). A systematic prediction of multiple drug-target interactions from chemical, genomic, and pharmacological data. *PLoS ONE*.

[19] Keiser M. J., Roth B. L., Armbruster B. N., Ernsberger P., Irwin J. J., Shoichet B. K. (2007). Relating protein pharmacology by ligand chemistry. *Nature Biotechnology*.

[20] Szklarczyk D., Santos A., Von Mering C., Jensen L. J., Bork P., Kuhn M. (2016). STITCH 5: Augmenting protein-chemical interaction networks with tissue and affinity data. *Nucleic Acids Research*.

[21] Yang H., Qin C., Li Y. H. (2016). Therapeutic target database update 2016: enriched resource for bench to clinical drug target and targeted pathway information. *Nucleic Acids Research*.

[22] Ye H., Ye L., Kang H. (2011). HIT: Linking herbal active ingredients to targets. *Nucleic Acids Research*.

[23] Szklarczyk D., Franceschini A., Wyder S. (2015). STRING v10: protein-protein interaction networks, integrated over the tree of life. *Nucleic Acids Research*.

[24] Assenov Y., Ramírez F., Schelhorn S.-E., Lengauer T., Albrecht M. (2008). Computing topological parameters of biological networks. *Bioinformatics*.

[25] Wang J., Vasaikar S., Shi Z., Greer M., Zhang B. (2017). WebGestalt 2017: A more comprehensive, powerful, flexible and interactive gene set enrichment analysis toolkit. *Nucleic Acids Research*.

[26] Cerami E., Gao J., Dogrusoz U. (2012). The cBio Cancer Genomics Portal: an open platform for exploring multidimensional cancer genomics data. *Cancer Discovery*.

[27] Gao J., Aksoy B. A., Dogrusoz U. (2013). Integrative analysis of complex cancer genomics and clinical profiles using the cBioPortal. *Science Signaling*.

[28] Keshava Prasad T. S., Goel R., Kandasamy K. (2009). Human protein reference database—2009 update. *Nucleic Acids Research*.

[29] Matthews L., Gopinath G., Gillespie M. (2009). Reactome knowledgebase of human biological pathways and processes. *Nucleic Acids Research*.

[30] Schaefer C. F., Anthony K., Krupa S. (2009). PID: the pathway interaction database. *Nucleic Acids Research*.

[31] Cerami E. G., Gross B. E., Demir E. (2011). Pathway Commons, a web resource for biological pathway data. *Nucleic Acids Research*.

[32] Fraser M., Sabelnykova V. Y., Yamaguchi T. N. (2017). Genomic hallmarks of localized, non-indolent prostate cancer. *Nature*.

[33] Robinson D., Van Allen E. M., Wu Y.-M. (2015). Integrative Clinical Genomics of Advanced Prostate Cancer. *Cell*.

[34] Beltran H., Prandi D., Mosquera J. M. (2016). Divergent clonal evolution of castration-resistant neuroendocrine prostate cancer. *Nature Medicine*.

[35] Baca S. C., Prandi D., Lawrence M. S. (2013). Punctuated evolution of prostate cancer genomes. *Cell*.

[36] Barbieri C. E., Baca S. C., Lawrence M. S. (2012). Exome sequencing identifies recurrent SPOP, FOXA1 and MED12 mutations in prostate cancer. *Nature Genetics*.

[37] Kumar A., Coleman I., Morrissey C. (2016). Substantial interindividual and limited intraindividual genomic diversity among tumors from men with metastatic prostate cancer. *Nature Medicine*.

[38] Taylor B. S., Schultz N., Hieronymus H. (2010). Integrative genomic profiling of human prostate cancer. *Cancer Cell*.

[39] Hieronymus H., Schultz N., Gopalan A. (2014). Copy number alteration burden predicts prostate cancer relapse. *Proceedings of the National Acadamy of Sciences of the United States of America*.

[40] Abeshouse A., Ahn J., Akbani R. (2015). The Molecular Taxonomy of Primary Prostate Cancer. *Cell*.

[41] Peifer M., Fernández-Cuesta L., Sos M. L. (2012). Integrative genome analyses identify key somatic driver mutations of small-cell lung cancer. *Nature Genetics*.

[42] Rudin C. M., Durinck S., Stawiski E. W. (2012). Comprehensive genomic analysis identifies SOX2 as a frequently amplified gene in small-cell lung cancer. *Nature Genetics*.

[43] Gao X. Q., Zhang W. D., Song S. Q., Wang L., Huang H. Y. (2002). Inhibitory effects of piroxicam on the transplanted sarcoma S180 of mice and its effect on the expression of COX-2, VEGF, FGF-2 and MVD. *Chinese Pharmacology Bulletin*.

[44] Sun J., Wen Q.-H., Li X. (2006). Comparison between antitumor effect and chemical constituents of Huanglian Jiedu decoction and that of serum containing Huanglian Jiedu decoction. *China Journal of Chinese Materia Medica*.

